# Overexpression of *MMP13* Is Associated with Clinical Outcomes and Poor Prognosis in Oral Squamous Cell Carcinoma

**DOI:** 10.1155/2014/897523

**Published:** 2014-10-23

**Authors:** Vui King Vincent-Chong, Iman Salahshourifar, Lee Peng Karen-Ng, Ming Yhong Siow, Thomas George Kallarakkal, Anand Ramanathan, Yi-Hsin Yang, Goot Heah Khor, Zainal Ariff Abdul Rahman, Siti Mazlipah Ismail, Narayanan Prepageran, Wan Mahadzir Wan Mustafa, Mannil Thomas Abraham, Keng Kiong Tay, Sok Ching Cheong, Rosnah Binti Zain

**Affiliations:** ^1^Oral Cancer Research and Coordinating Centre, Faculty of Dentistry, University of Malaya, 50603 Kuala Lumpur, Malaysia; ^2^Department of Oro-Maxillofacial Surgical and Medical Sciences, Faculty of Dentistry, University of Malaya, 50603 Kuala Lumpur, Malaysia; ^3^School of Pharmacy, Kaohsiung Medical University, Kaohsiung 807, Taiwan; ^4^Centre of Preclinical Science Studies, Faculty of Dentistry, Universiti Teknologi MARA, 40450 Shah Alam, Selangor Darul Ehsan, Malaysia; ^5^Department of Otorhinolaryngology, Faculty of Medicine, University of Malaya, 50603 Kuala Lumpur, Malaysia; ^6^Department of Oral and Maxillofacial Surgery, Hospital Kuala Lumpur, 50300 Kuala Lumpur, Malaysia; ^7^Department of Oral and Maxillofacial Surgery, Hospital Tengku Ampuan Rahimah, 41586 Klang, Malaysia; ^8^Department of Oral Surgery, Hospital Umum Kuching, 93586 Kuching, Sarawak, Malaysia; ^9^Oral Cancer Research Team, Cancer Research Initiatives Foundation, 47500 Selangor Darul Ehsan, Malaysia

## Abstract

Matrix metalloproteinase 13 (*MMP13*) plays a central role in the MMP activation cascade that enables degradation of the extracellular matrix and basement membranes, and it is identified as a potential driver in oral carcinogenesis. Therefore, this study aims to determine the copy number, mRNA, and protein expression of *MMP13* in oral squamous cell carcinoma (OSCC) and to associate these expressions with clinicopathological parameters. Copy number, mRNA, and protein expression analysis of *MMP13* were determined using real-time quantitative PCR and immunohistochemistry methods in OSCC samples. The correlations between *MMP13* expressions and clinicopathological parameters were evaluated, and the significance of *MMP13* as a prognostic factor was determined. Despite discrepancies between gene amplification and mRNA and protein overexpression rates, OSCC cases showed high amplification of *MMP13* and overexpression of *MMP13* at both mRNA and protein levels. High level of *MMP13* protein expression showed a significant correlation with lymph node metastasis (*P* = 0.011) and tumor staging (*P* = 0.002). Multivariate Cox regression model analysis revealed that high level of mRNA and protein expression of *MMP13* were significantly associated with poor prognosis (*P* < 0.050). Taken together, these observations indicate that the *MMP13* protein overexpression could be considered as a prognostic marker of OSCC.

## 1. Introduction

Oral cavity cancer is ranked as the sixth most common cancer worldwide, more than 90% of it being oral squamous cell carcinoma (OSCC) [1, 2]. Despite advances in diagnosis and treatment the survival rate still remains dismally low [3, 4]. Increased mortality rate could be attributed to late diagnosis and lack of specific biomarkers to predict tumor progression and prognosis of the patients [5, 6]. Hence, identifying specific biomarkers would pave the way for early detection and prognosis of OSCC.

We have recently detected several genomic copy number changes among OSCC cases [7]. Amplification at 11q23.3–q25 was found in 57% of OSCCs. The 11q22.2 region harbors a cluster of matrix metalloproteinases (MMPs) genes that play a pivotal role in tumor invasion and metastasis by degrading the extracellular matrix (ECM) [8]. The oncogenic role of MMP genes has been implicated in tumorigenesis and has widely been studied as potential biomarkers in various cancers, including OSCC [9]. Of these, overexpression of* MMP13* which is a collagenase appeared to be contributing to tumor cell invasion, metastasis, and poor prognosis [10]. Overexpression of this gene has been documented in numerous metastatic tumors such as head and neck SCC [11–13], vulvar SCC [14], laryngeal SCC [15], esophageal SCC [16], gastric cancer [17], malignant melanoma [18], bladder carcinoma [19], chondrosarcoma [20], colorectal carcinoma [21], breast carcinomas [22], and papillary thyroid carcinoma [23]. Product of* MMP13* digests collagen and other extracellular components; hence its overexpression could contribute in tumorigenesis via uncontrolled degradation of extracellular matrix components and basement membranes [10].

Based on our previous study [7], we hypothesized that amplification at 11q22.2 might be the possible explanation of* MMP13* overexpression and its tumorigenic role in OSCC. Multiple studies have reported overexpression of* MMP13* in head and neck SCC (HNSCC) [11–13, 24–26]. However there is paucity in research regarding the clinical outcomes of* MMP13* protein expression and its prognostic value in OSCC due to greater heterogeneity and aggressive features of OSCC as compared to other subsets of HNSCC [3, 27]. Hence, we further explored this gene at DNA, mRNA, and protein levels on independent samples to elucidate its potential role in tumorigenesis of OSCC and its correlation with clinical and survival characteristics in OSCC patients.

## 2. Materials and Methods

### 2.1. Samples Selection

We recruited 44, 68, and 103 independent OSCC samples for evaluation of DNA copy number, mRNA, and protein expression of* MMP13* gene, respectively. Forty-four DNA samples extracted from snap-frozen OSCC tissues were used for copy number analysis. Sections were stained using hematoxylin and eosin (H&E) and tumor cell percentage was gauged under microscope by two oral pathologists. In addition, cDNA of 68 OSCC and three normal mucosal samples were included for quantitation of the mRNA expression using real-time PCR. There were 21 OSCC samples overlapped between both copy number and mRNA expression analysis, 25 OSCC samples overlapped between both mRNA expression analysis and protein expression analysis, and 18 OSCC samples overlapped between both copy number analysis and protein expression analysis.

Immunohistochemical (IHC) analysis was performed on formalin fixed paraffin embedded (FFPE) tissues and frozen tissue sections. The FFPE tissues included 20 oral dysplastic lesions (ODLs), 5 normal oral mucosal tissues and 77 OSCC samples. The frozen tissue sections consisted of 26 OSCC samples. The FFPE samples were obtained from the archives of Oral Pathology Diagnostic and Research laboratory at the University of Malaya. The OSCC tissue specimens were derived from the tongue (excluding the base of the tongue), buccal mucosa, gum, palate, floor of mouth, and lip (C00-06). All the tumor tissues were surgical excision specimens. The normal samples were obtained from normal oral mucosa adjacent to impacted wisdom teeth during surgical removal of the impacted teeth. All the frozen tissues were immediately snapped frozen in liquid nitrogen. Frozen tissue samples and sociodemographic and clinicopathologic data of OSCC samples were obtained from the Malaysian Oral Cancer Database and Tumor Bank System (MOCDTBS) managed by the Oral Cancer Research and Coordinating Centre, University of Malaya (OCRCC, UM) [28]. The American Joint Committee on cancer staging criteria was used for tumor staging [29]. All OSCC patients recruited in this study were treated based on pTNM staging that included surgery alone and a combination of surgery with radiotherapy and surgery with radiotherapy and chemotherapy. Written informed consent was obtained before sample collection. The specimens were collected, stored, and used later for this study. This study was approved by Medical Ethics Committee, Faculty of Dentistry, University of Malaya [MEC number DFOP1108/0083(L)].

### 2.2. Copy Number Analysis by the TaqMan PCR Assay

DNA was extracted from normal samples/tumor tissues with ≥70% tumor cell content using DNEasy Blood & Tissue kit (Qiagen, Hilden, Germany) according to manufacturers' protocol. Copy number analysis was performed on 44 OSCCs according to the manufacturer's protocol as previously described [7]. Briefly, each gDNA was analyzed in quadruplicate by duplex TaqMan real-time polymerase chain reaction assays. The gDNA from 2 healthy volunteers (female and male) and 2 normal oral mucosa tissues served as calibrator controls. Copy number analysis was done using* MMP13* TaqMan Copy Number Assay (Hs01829774_cn) (Applied Biosystems, Foster City, CA, USA). PCR was done in a total volume of 20 *μ*L consisting of 4 *μ*L of genomic DNA (5 ng/*μ*L), 10 *μ*L of 2x TaqMan Genotyping Master Mix (Applied Biosystems, Foster City, CA, USA), 1 *μ*L of 20x TaqMan Copy number assay, 1 *μ*L of 20x TaqMan copy number reference assay (RNAse P), and 4 *μ*L of nuclease free water. Quantitative PCR was performed on an ABI 7500 Fast Real Time PCR System (Applied Biosystems, Foster City, CA, USA) using the manufacturer's PCR conditions as follows: initial denaturation at 95°C for 10 minutes followed by 40 cycles of denaturation for 15 seconds at 95°C and annealing for 60 seconds at 60°C.

The values of copy number for each sample were normalized using RNAase P as a reference control with 2 copies in the human genome. Copy number was quantified using the equation 2 × (2 − ΔΔCT), comparative CT (ΔΔCT) relative quantitation method [30]. Target and reference assays that were used for copy number calculation were derived from the mean of quadruplicate, RNase P, and the calibrator samples. The calculated relative quantity was multiplied by a base copy number of 2 to obtain the copy number value. The values less than one and more than 2.5 were considered as deletion and amplification, respectively [31].

### 2.3. mRNA Expression of* MMP13* Using qRT-PCR

RNA extraction was done on normal samples/tumor tissues with ≥70% tumor cell content using RNeasy Micro kit (Qiagen, Hilden, Germany) according to manufacturers' protocol. The integrity of RNA was tested using Agilent Bioanalyzer-2100 (Agilent, Palo Alto, CA, USA). Reverse transcription of total RNA was done using High Capacity cDNA reverse transcription kit (Applied Biosystems, Foster City, CA, USA). The cDNA of each sample was obtained in triplicate and the gene expression of* MMP13* was performed using 7500 Fast Real-Time PCR System (Applied Biosystems, Foster City, CA, USA). TaqMan Gene Expression Assay was carried out for* MMP13* (Hs00233992_m1) according to the manufacturer's protocol (Applied Biosystems, Foster City, CA, USA). The relative quantification/fold change (RQ) was calculated based on the 2 − ΔΔCT method using 7500 Fast System SDS Software 1.3.1 (Applied Biosystems, Foster City, CA, USA). The GAPDH gene was used as endogenous control and the cDNA from normal oral mucosa tissues (RQ = 1) was utilized for normalization of test samples.

## 3. Tissue Microarray

Tissue microarray (TMA) of 1.0 mm core size was constructed as described previously [32] using a semiautomatic Tissue Arrayer Minicore (Alphelys, SAS, France). All 77 OSCC FFPE blocks and the respective 5 *μ*m H&E stained slides were selected to identify and mark out the representative tumor areas by 2 oral pathologists independently. Approximately, 3–6 cores from the selected areas of donor blocks were transferred to the recipient paraffin blocks. The completed recipient paraffin blocks also known as TMA were incubated overnight at 37°C and 4 *μ* thick sections were sectioned on poly-lysine slides.

### 3.1. Immunohistochemistry and Scoring System

IHC was performed on 4 *μ*m thick FFPE sections using the Envision technique, Dako Real EnVision Detection System and Peroxidase/DAB+ (Dako Corporation, Carpinteria, CA, USA) according to the manufacturer's protocol. Briefly, FFPE sections were deparaffinized in Xylene and rehydrated in ethanol series. Antigen retrieval was carried out using an electric pressure cooker (110°C, 20 minutes) in 10 mM citrate buffer (pH 6.0). The sections were immersed in blocking solution (Dako Corporation, Carpinteria, CA, USA) for 10 min at room temperature followed by washing with Phosphate-buffered saline (pH 7.4) plus 0.1% Tween 20 for blocking the endogenous peroxidase activity. The sections were then incubated with 8 *μ*g/mL of monoclonal anti-*MMP13* (MAB511, R&D Systems, Inc, Heidelberg, Germany) overnight at 4°C for FFPE sectioned and one hour at room temperature for frozen tissue sectioned. After washing with PBS buffer, sections were incubated with the peroxidase labeled secondary antibody from the Envision kit for 45 minutes for the immunoreactivity performances. Finally, sections were stained with 3′3 diaminobenzidine substrate chromogen (Dako Corporation, Carpinteria, CA, USA), counterstained with Mayer's hematoxylin, dehydrated, and mounted.

Digitalized immunostained TMA spots were analyzed and scored by 2 oral pathologists independently based on semiquantitative scoring system using TMA software module 1.15.2 (3DHISTECH, Budapest, Hungary). The intensity scores were quantified using the following scores: negative = 0; weak = 1; moderate = 2; and strong = 3. The proportion of immunopositive cells was quantified as follows: 0 = negative; 1 = <10%; 2 = 11–50%; 3 = 51–80%; and 4 = ≥80% of positive cells. The final immunoreactive score was determined by multiplying the intensity and the proportion scores of the stained cells to obtain an immunoreactive score ranging from 0 to 12 [33, 34]. Cores with discrepant scores were discussed by both pathologists to achieve a consensus to derive the final score. The mean of consolidated immunoreactive scores for each case was recorded.

### 3.2. Selection of Cutoff Score for* MMP13* Protein Expression

The clinicopathological parameters were first dichotomized as follows: lymph node metastasis (no versus yes), tumor staging (early versus advanced), tumor sizes (T1 and T2 versus T3 and T4), and survival status (alive versus dead). Receiver operating characteristic (ROC) curve analysis was used to determine the best cutoff score for* MMP13* protein expression to each of dichotomized clinicopathological parameters using 0, 1 criterion [35]. For* MMP13* immunoreactive scoring, the sensitivity and specificity of each score were plotted to generate various ROC curves. The score which was closest to the point with maximum sensitivity and specificity was selected as the cutoff value. The immunoreactive scores were divided into high and low* MMP13* expression where low expression was the scores below or equal to the cutoff value, while high expression was the scores above the cutoff value.

### 3.3. Statistical Analysis

Copy number alterations, mRNA, and protein expression level of* MMP13* were compared between tumor and normal tissues using the Mann-Whitney *U* test. The copy number of* MMP13* was classified into two groups, amplification (>2.5 copies) and nonamplification (≤2.5 copies). Gene expression of* MMP13* was classified into two groups, high and low, with a cutoff value based on the 75th percentile of the respective relative quantitative (RQ) values. A receiver operating characteristics (ROC) curve was used to determine the best cut-off point based on the immunoreactive scores of the* MMP13* for specificity and sensitivity. Correlation between copy number and gene expression levels of* MMP13* was assessed via Spearman correlation analysis. Associations between the copy number, mRNA, and protein expression of* MMP13* and the clinicopathological parameters were analyzed by chi square test (or Fisher exact test where appropriate). Survival curves were plotted and compared by the log rank tests using the Kaplan-Meier analysis. In addition, Cox regression analysis was conducted to evaluate the* MMP13* expression as an independent prognostic factor. All statistical analyses were performed using the SPSS statistical package (SPSS version 12.0, Chicago, IL, USA) and the *P* value < 0.05 was considered significant.

## 4. Results

### 4.1. Definition of Cutoff Score for* MMP13* Protein Expression in OSCC

**Figure 1:**
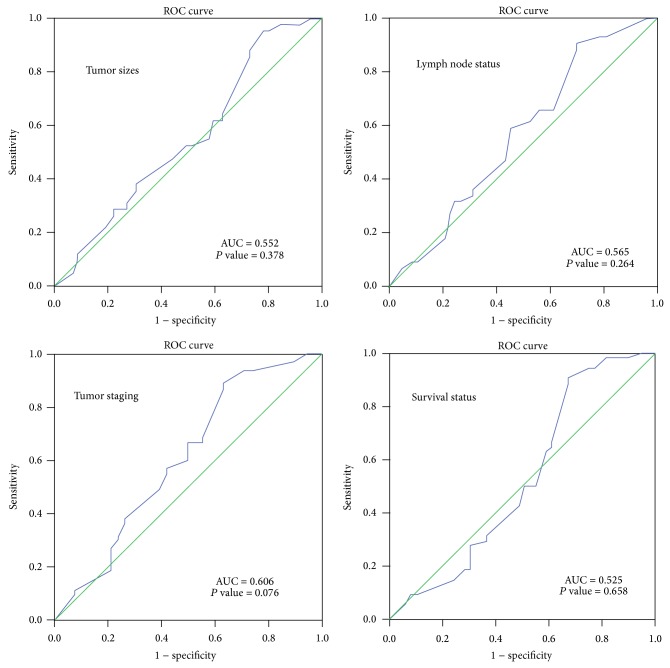
Determination the cutoff value of* MMP13* expression in OSCC by receiver operating characteristic (ROC) curves. The clinicopathological parameters including lymph node metastasis, tumor staging, tumor sizes and survival status, the sensitivity, and 1 − specificity were plotted. The areas under curve (AUC) and the *P* value were indicated.

ROC curve analysis was performed based on the results of IHC evaluation. Results showed that ROC curve analysis for tumor staging has the shortest distance from the curve to the point (0.0, 1.0) (Table 1; Figure 1). Hence, cutoff value for tumor staging was selected. The cutoff score for low* MMP13* expression was set to <3.50 and the counterpart as high* MMP13* expression.

### 4.2. *MMP13* Gene Copy Number, mRNA, and Protein Expression in OSCC

**Figure 2:**
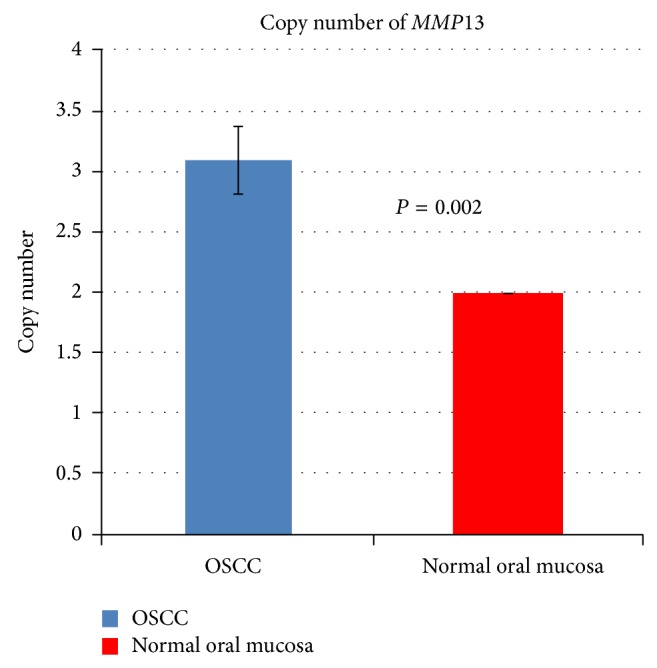
Copy number alterations of* MMP13* between OSCC and normal oral mucosa. The copy number alterations of* MMP13* between OSCC and normal oral mucosa tissues were statistically different (*P* = 0.002) with an average of 3.09 copies. The copy number for normal oral mucosa (NT) of* MMP13* was 2 due to presentation as two diploid copies as reference control.

**Figure 3:**
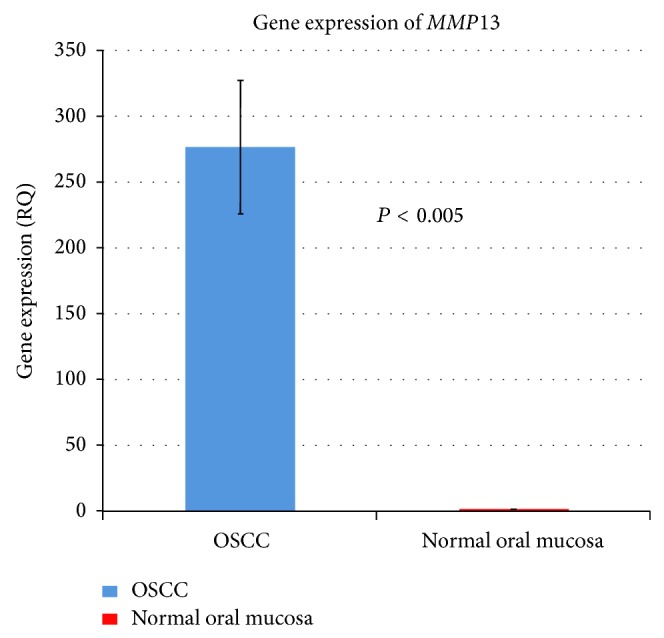
The gene expression level (RQ) of* MMP13* in OSCC samples based on the fold change expressed as an average of 68 OSCC samples. Expression level of* MMP13* was RQ = 276.28 while the gene expression between OSCC and normal mucosa was statistically different (*P* < 0.005). The RQ for normal tissue (NT) of* MMP13* was 1 due to the normalization.

Amplification of* MMP13* was identified in 59.1% of the OSCC samples (26 out of 44) with an average copy number of 3.09 ± 1.81 (Figure 2). In line with this the* MMP13* mRNA was found to be expressed at a high level in 95.59% of the OSCC samples (65 out of 68) with an average gene expression fold change of RQ = 276.28 (Figure 3). Spearman's correlation coefficient showed a nonsignificant correlation between copy number and gene expression of* MMP13* (*n* = 21, *r* ^2^ = 0.237, *P* = 0.302), between copy number and protein expression of* MMP13* (*n* = 18, *r* ^2^ = 0.125, *P* = 0.621), and between gene expression and protein expression of* MMP13* (*n* = 23, *r* ^2^ = 0.378, *P* = 0.062).

**Figure 4:**
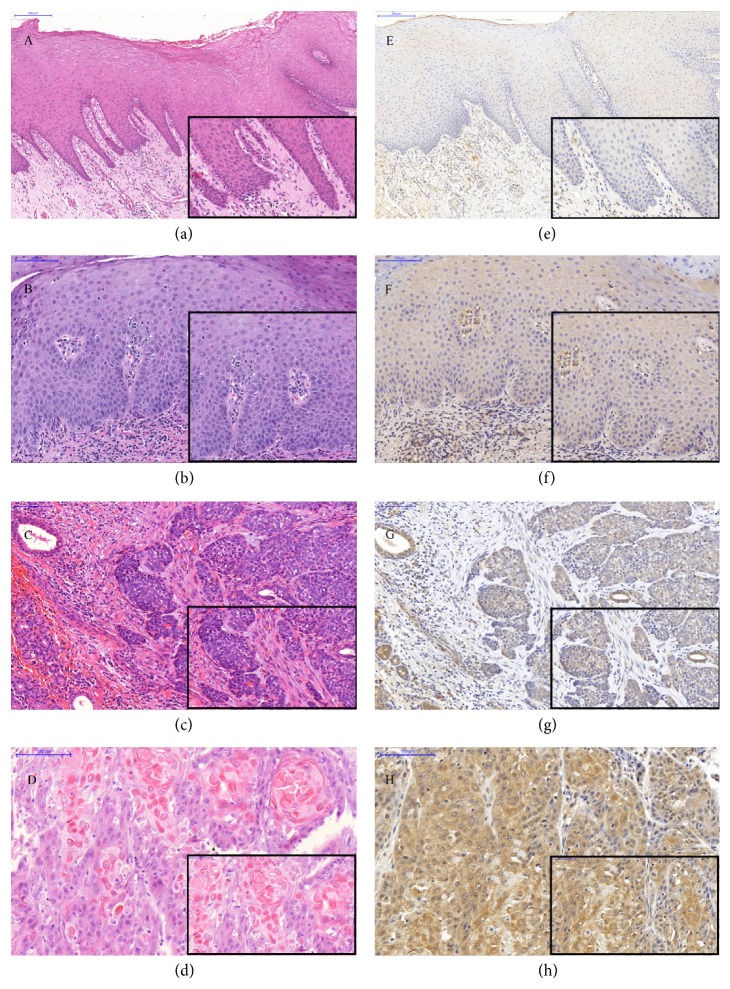
Immunohistochemistry of* MMP13*. Normal oral mucosa (a) H&E stain (magnification 400x and 1600x); (e) anti-*MMP13* antibody immunostain was negative in the normal oral mucosa (magnification 400x and 1600x). Dysplastic oral tissue (b) H&E stain (magnification 800x and 1600x); (f) anti-*MMP13* antibody showed weak to moderate immunostaining in the cytoplasm of the dysplastic epithelial cells (magnification 800x and 1600x). OSCC (c and d) H&E stained (magnification 800x and 1600x); (g) anti-*MMP13* antibody immunostaining showed low expression and (h) high expression in the cytoplasm of the epithelial tumor cells (magnification 800x and 1600x). All the oral tissues showed moderate anti-*MMP13* antibody immunostaining of the stroma and inflammatory cells in the microenvironment.

In IHC analysis of* MMP13* protein, the epithelial cells of normal oral mucosal tissues showed a negative staining. A weak to moderate staining was seen in the cytoplasm of the epithelial cells of the basal and spinous layers in dysplastic oral mucosa. More than 75% of OSCCs displayed a strong staining in the cytoplasm of epithelial tumor cells. All the normal, dysplastic, and OSCC tissue samples demonstrated moderate* MMP13* immunostaining of the stromal compartment including the inflammatory cells. The expression of* MMP13* protein was statistically different between OSCC and normal oral mucosal tissues (*P* < 0.05) in contrast to OSCC and ODLs (Figure 4).

### 4.3. Association of* MMP13* Gene Copy Number, mRNA, and Protein Expression with Clinicopathologic Parameters

Change in copy number of* MMP13* gene was found to be statistically significant between OSCC and normal oral mucosal tissues (*P* = 0.002). However, there was no significant association between copy number alterations and clinicopathologic factors. Expression of* MMP13* mRNA was significantly higher in OSCCs compared with normal oral mucosa samples (*P* < 0.005), but it had no significant association with clinicopathologic factors. In contrast, high expression of* MMP13* protein was significantly correlated with lymph node metastasis (*P* = 0.011), tumor staging (0.002), and a trend towards association with tumor sizes (T3 and T4, *P* = 0.063) (Table 2).

### 4.4. Significance of* MMP13* Gene Copy Number, mRNA, and Protein Expression as Prognostic Indicators

**Figure 5:**
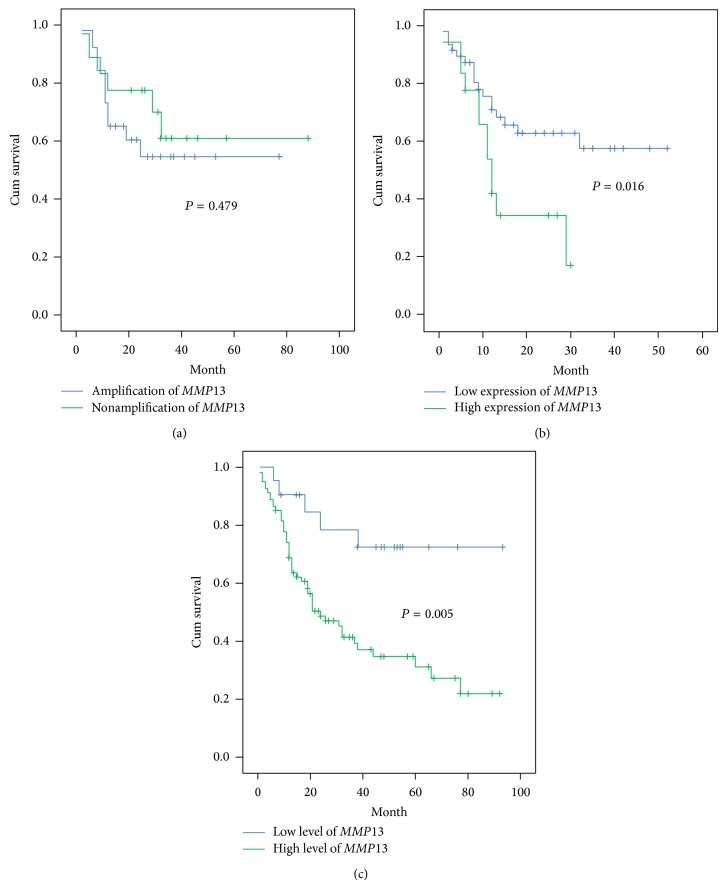
Overall survival curves were analyzed according to* MMP13* copy number (a), mRNA expression (b), and protein expression (c) using Kaplan-Meier estimate with log rank test.

The follow-up time for patients that were recruited for copy number analysis of* MMP13* ranged from 2 to 88 months (mean: 26.73 months, median: 24.5 months). Two-year survival rates for low and high copy number of* MMP13* were 70.0% and 55.19%, respectively. Results of Kaplan-Meier analysis showed no significant association between* MMP13* amplification and poor prognosis (*P* = 0.479) (Figure 5).

The follow-up time for patients that were used for analysis of* MMP13* mRNA expression ranged from 1 month to 52 months (mean: 17.71 months, median: 13.0 months). Three-year survival rates for low and high expression of* MMP13* were 57.76% and 17.45%, respectively. The* MMP13* mRNA expression showed significant correlation with poor prognosis (*P* = 0.016) in Kaplan-Meier analysis (Figure 5). In multivariate Cox regression analysis, the expression of* MMP13* mRNA remained as a significant prognostic factor for survival after adjustment for age, gender, risk habits, and clinicopathologic parameters (tumor sites, lymph node metastasis, and tumor staging) which are the common confounding factors in OSCC (HRR = 2.23, 95% CI 1.015–4.896, *P* = 0.046) (Table 3).

For* MMP13* protein expression, the follow-up time for patients ranged from 1 month to 92 months (mean: 29.13 months, median: 20.5 months). Three-year survival rate for the high and low expression of* MMP13* protein was 34.73% and 72.38%, respectively. Results of the five-year survival rate analysis demonstrated a significant association between positive* MMP13* protein expression and poor prognosis (*P* = 0.005) (Figure 5).

After adjustment for selected sociodemographic (age, gender, and risk habits) and clinicopathological parameters (tumor subsite, tumor differentiation, and pattern of invasion), positive* MMP13* expression remained a significant prognostic factor for overall survival of OSCC (HRR = 3.850, 95% CI 1.234–12.010, *P* = 0.020, data not shown). Positive* MMP13* expression showed a considerable trend as an independent prognostic factor towards unfavorable overall survival after adjustment with other clinicopathological parameters such as tumor subsites, lymph node metastasis, tumor staging, pattern of invasion, and tumor differentiation (HRR = 2.84, 95% CI 0.922–8.768, *P* = 0.069) (Table 4).

## 5. Discussion

Despite several studies that have demonstrated the overexpression of* MMP13* mRNA and protein expression among OSCCs and head and neck SCCs [11–13, 24–26, 36–38], the reason for overexpression and its role in pathogenesis of OSCC remained unanswered. Copy number alterations are widely accepted as one of the major drivers in cancer mainly by altering the gene expression levels [39]. Amplification in 11q22.2 which harbors the* MMP* genes was a frequent finding in our previous study [7]. Hence, we postulated that the pathogenic role of* MMP13* overexpression could be linked to copy number changes at this region. Therefore, the role of this gene in pathogenesis of OSCC was explored using independent set of samples at DNA, mRNA, and protein levels as independent set of samples would draw a stronger conclusion for biomarker discovery in cancer [40]. In line with our previous study [7], amplification of* MMP13* gene was common and was found in 59.1 of cases while overexpression at both mRNA and protein levels was more frequent and found in 95.6% and 79.6% of patients, respectively. Consistent changes at DNA, mRNA, and protein levels of* MMP13* on independent set of samples reflect that gene amplification could be one of the possible mechanisms for* MMP13* overexpression. However, gene amplification may increase gene expression at both mRNA and protein levels but concurrent changes in mRNA and protein levels do not correlate in most of the cases mainly due to the regulatory controls at different levels [41]. Hence, a trend of correlation would be expected and only a small percentage of transcriptional changes would correspond to similar protein expression changes. Thus, investigation of mRNA and protein expression even on the same samples may not guarantee a statistical correlation between these events as seen in Yamamoto et al. [41]. In the current study, similar trend of overexpression at different levels on independent set of samples could be considered as a positive correlation despite insignificant statistical correlation. In other words, identifying overexpression of* MMP13* mRNA in a high percentage of patients reflects that* MMP13* protein could be an appropriate potential biomarker for further analysis among OSCCs as a trend toward significant correlation was found between mRNA and protein expression (*r* = 0.378, *P* = 0.062).

*MMP13* protein was highly expressed in epithelial cells of OSCCs as compared to normal oral mucosal epithelial cells. This was in concordance with the statistical difference that was found in copy number changes at DNA level between OSCCs and normal mucosa. Therefore, overexpression of* MMP13* could be the consequence of amplification. In addition, increased expression of* MMP13* protein from epithelial cells of normal mucosa as compared to OSCC reflects the important role of this gene in progression to OSCC. Our results were consistent with the previously reported evidence that was conducted on ODLs and OSCCs [36]. Hence,* MMP13* protein might be considered as a useful biomarker for ODLs with a risk of malignant transformation. However, the sample size of ODLs was small to draw a strong conclusion; hence further investigation will be needed.

Despite lack of significant association between copy number and mRNA expression of* MMP13* with clinicopathologic parameters, overexpression of* MMP13* mRNA was associated with poor prognosis and remained as an independent prognostic factor. Similar evidence has been reported on esophageal SCC [37]. Our literature review yielded only two investigations that have been conducted on the prognostic value of* MMP13* in OSCCs till date [36, 38]. The first study did not observe any association between overexpression of* MMP13* and clinical outcome as well as poor survival [36]. The second study which mainly focused on oral tongue SCC found a significant role for* MMP13* as a prognostic marker [38]. In the current study, overexpression of* MMP13* protein showed significant association with advanced staging and lymph node metastasis. This observation reflects the proteolytic activity of* MMP13* in degradation of the ECM and basement membrane which promotes the tumor progression and invasion in OSCC. To date, there has been no extensive study on the relationship between* MMP13* protein expression and lymph node metastasis in OSCC. These findings provide further support that* MMP13* is involved in OSCC invasion and metastasis. In addition, it showed association with poor prognosis and remained as an independent prognostic factor after adjusting with selected clinicopathological parameters (tumor subsites and tumor differentiation) but the prognostic value of* MMP13* was attenuated after controlling with lymph node status and tumor staging. This implies that significance of using* MMP13* as a prognostic marker may be more pronounced after taking into account the patient's lymph node status and tumor stage.

Taken together, the overexpression of* MMP13* was identified as an independent prognostic marker for OSCC at both mRNA and protein expression levels. In addition, increased expression of* MMP13* protein in ODLs and OSCC as compared to normal oral mucosa and its correlation with advanced stage and lymph node metastasis of OSCC provide further evidence for its role in genesis and progression of OSCC. Further investigations regarding the interaction of* MMP13* with other potential genes or environmental risk factors would shed light on the complex role of this gene in pathogenesis of OSCC.

## Figures and Tables

**Table 1:** Area under the receiver operating characteristic curve (AUC) for each clinicopathological feature.

Clinicopathological parameters	AUC (95% CI)	*P* value
Lymph node metastasis (yes versus no)	0.565 (0.453–0.677)	0.264
Tumor staging (advanced versus early)	0.606 (0.486–0.725)	0.076
Tumor sizes (T1 and T2 versus T3 and T4)	0.552 (0.439–0.664)	0.378
Survival (death versus alive)	0.525 (0.409–0.642)	0.658

**Table 2:** Association of *MMP13* protein expression with clinicopathological parameters.

Variables	Category	Number of patients (%)	*MMP13* expression (*n*, %)		*P* value
			Low level of expression	High level of expression	
		103	21 (20.4)	82 (79.6)	

Total					

Gender	Male	35 (34.0)	10 (28.6)	25 (71.4)	0.139
	Female	68 (66.0)	11 (16.2)	57 (83.8)	

Age (years)	<45	11 (10.7)	3 (27.3)	8 (72.7)	0.691
	≥45	92 (89.3)	18 (19.6)	74 (80.4)	

Smoking	No	81 (78.6)	15 (18.5)	66 (81.5)	0.380
	Yes	22 (21.4)	6 (27.3)	16 (72.7)	

Drinking	No	71 (68.9)	13 (18.3)	58 (81.7)	0.435
	Yes	32 (31.1)	8 (25.0)	24 (75.0)	

Betel quid chewing	No	49 (47.6)	10 (20.4)	39 (79.6)	0.996
	Yes	54 (52.4)	11 (20.4)	43 (79.6)	

Tumor site	Non-tongue∗	68 (66.0)	14 (20.6)	54 (79.4)	0.944
	Tongue	35 (34.0)	7 (20.0)	28 (80.0)	

Tumor size∗∗	T1-T2	59 (58.4)	16 (27.1)	43 (72.9)	0.063
	T3-T4	42 (41.6)	5 (11.9)	37 (88.1)	

Lymph node metastasis∗∗	Negative	57 (56.4)	17 (29.8)	10 (70.2)	**0.011**
	Positive	44 (43.6)	4 (9.1)	40 (90.9)	

pTNM staging∗∗	Early stage	38 (37.6)	14 (36.8)	24 (63.2)	**0.002**
	Advanced stage	63 (62.4)	7 (11.1)	56 (88.9)	

Pattern of invasion∗∗	Cohesive	13 (15.5)	5 (38.5)	8 (61.5)	0.140
	Non-cohesive	71 (84.5)	13 (18.3)	58 (81.7)	

Differentiation∗∗	Well	45 (44.1)	11 (24.4)	34 (75.6)	0.392
	Poor and Moderate	57 (55.9)	10 (17.5)	47 (82.5)	

^*^Buccal mucosa, gingiva, lip, floor of mouth, palate.

∗∗Data missing.

Significant *P* values were highlighted in bold.

**Table 3:** Multivariate cox regression model analysis of *MMP13* mRNA expression in OSCC overall survival.

Variables	Category	Number of patients (%)	Multivariate Logistic regression∗∗		
			OR	95% CI	*P* value
Total		68			

mRNA expression of *MMP13 *	Low	50 (73.5)	1.00^†^	1.015–4.896	**0.046**
	High	18 (26.5)	2.23		

Gender	Male	24 (35.3)	1.00^†^	0.388–2.806	0.933
	Female	44 (64.7)	1.043		

Age (years)	<45	12 (17.6)	1.00^†^	0.397–3.009	0.864
	≥45	56 (82.4)	1.092		

Smoking	No	44 (64.7)	1.00^†^	0.240–2.071	0.524
	Yes	24 (35.3)	0.704		

Drinking	No	51 (75.0)	1.00^†^	0.391–2.341	0.922
	Yes	17 (25.0)	0.956		

Betel quid chewing	No	40 (58.8)	1.00^†^	0.652–3.718	0.319
	Yes	28 (41.2)	1.557		

Tumor site	Non-tongue∗	38 (55.9)	1.00^†^	0.516–2.933	0.640
	Tongue	30 (44.1)	1.230		

Lymph node metastasis	Negative	33 (48.5)	1.00^†^	1.028–20.275	**0.046**
	Positive	35 (51.5)	4.565		

pTNM Staging	Early	22 (32.4)	1.00^†^	0.339–13.469	0.419
	Advanced	46 (67.6)	2.137		

CI: confidence interval.

∗Buccal mucosa, gingiva, lip, floor of mouth, palate.

^†^Reference category.

Significant *P* values were highlighted in bold.

∗∗Multivariate logistic regression analysis was applied to adjust the confounders [age, gender, risk habits (cigarette smoking, betel quid chewing, and alcohol drinking)], and clinicopathologic parameters [tumor sites, lymph node metastasis, and pathological tumor staging].

**Table 4:** Multivariate cox regression model analysis of *MMP13* protein expression in OSCC overall survival.

Variables	Category	Number of patients (%)	Multivariate Logistic regression∗∗		
			OR	95% CI	*P* value
Total		103			

Protein expression of *MMP13 *	Low	21 (20.4)	1.00^†^	0.922–8.768	0.069
	High	82 (79.6)	2.84		

Gender	Male	35 (34.0)	1.00^†^	0.366–2.530	0.938
	Female	68 (66.0)	0.96		

Age (years)	<45	11 (10.7)	1.00^†^	0.127–2.997	0.548
	≥45	92 (89.3)	0.62		

Smoking	No	81 (78.6)	1.00^†^	0.150–2.017	0.367
	Yes	22 (21.4)	0.55		

Drinking	No	71 (68.9)	1.00^†^	0.376–1.656	0.531
	Yes	32 (31.1)	0.79		

Betel quid chewing	No	49 (47.6)	1.00^†^	0.195–1.178	0.109
	Yes	54 (52.4)	0.48		

Tumor site	Non-tongue∗	68 (66.0)	1.00^†^	0.306–1.785	0.502
	Tongue	35 (34.0)	0.74		

Lymph node metastasis∗∗∗	Negative	57 (56.4)	1.00^†^	0.771–4.188	0.175
	Positive	44 (43.6)	1.80		

pTNM staging∗∗∗	Early	38 (37.6)	1.00^†^	0.662–4.683	0.257
	Advanced	63 (62.4)	1.76		

Pattern of invasion∗∗∗	Cohesive	13 (15.5)	1.00^†^	0.839–10.374	0.09
	Noncohesive	71 (84.5)	2.95		

Differentiation∗∗∗	Well	45 (44.1)	1.00^†^	0.258–1.009	0.05
	Moderate and poor	57 (55.9)	0.51		

CI: confidence interval.

∗Buccal mucosa, gingiva, lip, floor of mouth, palate.

^†^Reference category.

∗∗Multivariate logistic regression analysis was applied to adjust the confounders [age, gender, risk habits (cigarette smoking, betel quid chewing and alcohol drinking)] and clinico-parameters [tumor subsites, lymph node metastasis, tumor staging, pattern of invasion and pathological tumor differentiation].

∗∗∗Data missing.
